# Improvement of Must Fermentation from Late Harvest cv. Tempranillo Grapes Treated with Pulsed Light

**DOI:** 10.3390/foods10061416

**Published:** 2021-06-18

**Authors:** Carlos Escott, Carmen López, Iris Loira, Carmen González, María Antonia Bañuelos, Wendu Tesfaye, José Antonio Suárez-Lepe, Antonio Morata

**Affiliations:** 1 enotecUPM, Chemistry and Food Technology Department, Escuela Técnica Superior de Ingeniería Agronómica, Alimentaria y de Biosistemas, Universidad Politécnica de Madrid, Avenida Puerta de Hierro 2, 28040 Madrid, Spain; carmen.lopez@upm.es (C.L.); iris.loira@upm.es (I.L.); carmeng.chamorro@upm.es (C.G.); wendu.tesfaye@upm.es (W.T.); joseantonio.suarez.lepe@upm.es (J.A.S.-L.); antonio.morata@upm.es (A.M.); 2 Biotechnology and Vegetal Biology Department, Escuela Técnica Superior de Ingeniería Agronómica, Alimentaria y de Biosistemas, Universidad Politécnica de Madrid, Avenida Puerta de Hierro 2, 28040 Madrid, Spain; mantonia.banuelos@upm.es

**Keywords:** pulsed light, anthocyanins, non-*Saccharomyces*, Tempranillo, AFM, lactic acid, volatiles

## Abstract

Pulsed light irradiation is a nonthermal technology currently used for the elimination of pathogens from a diverse range of food products. In the last two decades, the results obtained using PL at laboratory scale are encouraging wine experts to use it in the winemaking industry. PL can reduce native yeast counts significantly, which facilitates the use of starter cultures, reducing SO_2_ requirements at the same time. In this experimental set up, Tempranillo grapes were subjected to pulsed light treatment, and the fermentative performance of non-*Saccharomyces* yeasts belonging to the species *Schizosaccharomyces pombe*, *Lachancea thermotolerans*, *Torulaspora delbrueckii*, *Metschnikowia pulcherrima* and *Hanseniaspora vineae* was monitored in sequential fermentations against spontaneous fermentation and pure culture fermentation with the species *Saccharomyces cerevisiae*. The experimental analyses comprised the determination of anthocyanin (High performance liquid chromatography with photodiode array detector—HPLC-DAD), polyphenol index and colour (Ultraviolet-visible spectroscopy—UV-Vis spectrophotometer), fermentation-derived volatiles (Gas chromatography with flame ionization detector—GC-FID), oenological parameters (Fourier transform Infrared spectroscopy—FT-IR) and structural damage of the skin (atomic force microscopy—AFM). The results showed a decrease of 1.2 log CFU/mL yeast counts after pulsed light treatment and more rapid and controlled fermentation kinetics in musts from treated grapes than in untreated samples. The fermentations done with treated grapes allowed starter cultures to better implant in the must, although a larger anthocyanin loss (up to 93%) and an increase in hue values (1 unit) towards more yellow hues were observed for treated grapes. The development of biomass was larger in musts from treated grapes. The profile of volatile compounds and oenological parameters reveals that fermentations carried out with untreated grapes are prone to deviations from native microbiota (e.g., production of lactic acid). Finally, no severe damage on the skin was observed with the AFM on treated grapes.

## 1. Introduction

Pulsed light irradiation (PL) is an emerging nonthermal technology currently used for the sanitation of food products [1,2,3]. It is considered nonthermal since the temperature of the treated meal does not experience a significant increase. PL irradiation, as well as other nonthermal technologies, has been used effectively in the reduction of spoilage microbial populations [4,5], and this may eventually reduce the risk of the accumulation of biogenic amines, especially from lactic acid bacteria [6], as a tool to ensure quality control. PL comprises irradiation from the near-infrared, visible and ultraviolet fractions (UV-A, UV-B and UV-C) in the electromagnetic spectrum. The effectiveness of this nonthermal technology is based on the damage produced to cellular wall structures and at the DNA level by the ultraviolet fraction from the pulses [7,8], especially the fraction UV-C. The damages include the formation of dimers from adjacent thymines, inhibition of DNA replication and single and double-stranded breaks of DNA chains. The UV fraction can eliminate wild microbial populations located in the pruina of grape skins as previously reported [9]. 

There is currently little research on the PL treatment of grapes for winemaking. Documented treatments include 5 to 10 pulses and fluences up to 1.8 J/cm^2^ [9]. Treatments in white wine, for the reduction of spoilage yeasts, have used larger fluence values (>10 J/cm^2^) [10]. In comparison, in the treatment of other food matrices with PL, the fluence can reach 14 J/cm^2^ and 16 J/cm^2^ for avocado and raspberry, respectively [11]. The fluence may vary in function of the power of the apparatus and the duration of the pulses. The energy is kept in the capacitors before it is released in the ultrashort periods of time [7]. 

The use of PL as a pretreatment in vinification would allow winemakers to use starter cultures to better control the fermentation process [11,12]. PL would reduce the wild native yeasts, mainly non-*Saccharomyces* yeasts which are larger in number than the *Saccharomyces* genus, stopping spontaneous fermentations from taking over and allowing selected strains or starter cultures to implant faster. Non-*Saccharomyces* yeasts, especially the genus *Hanseniaspora*, are particularly present in higher amounts in the pruina of the grapes and are responsible for the start of the fermentation [13].

PL can also be used in other winemaking processes. The irradiation emitted is also capable of reducing spoilage yeasts in wines. Such is the case of white wine inoculated with *Brettanomyces bruxellensis*. The treatment with PL produced up to a 2-log reduction with a fluence of 10.7 J/cm^2^ [10]. Nonetheless, the use of PL for this purpose may induce changes in the quality of the wines as a result of the modification of the organoleptic parameters. Other technologies, such as pulsed electric fields and UV-C irradiation, have also demonstrated inactivating spoilage yeast from the species *B. bruxellensis* [14].

Other effects attributed to the use of PL pretreatments in other food matrices are: (1) the preservation of quality properties, as the energy associated with this technology does not modify the phenolic composition and antioxidant properties [15], and the preservation of organoleptic attributes associated with food matrices [16] and (2) the increased extraction of phenolics and anthocyanin’s fractions [17,18], the effect of which is an enhanced colour expression. In the winemaking process, the colour improvement attributed to PL goes together with appropriate practices and, mainly, a proper maceration, which is a physicochemical process in which mechanical operations, temperature and aeration are involved [19]. 

The principal challenge found for PL to be used for musts in the winemaking process is low transparency and suspended solids [20]. Suspended solids would reduce the penetration of the irradiation. For the UV fraction to be effective in red wine must, due to the high UV absorbance of nonclarified liquids, the width of the piping should be thin [21]. An arrangement of lamps and pipes is to be designed to achieve the microbial reduction needed. 

In this experimental work, microfermentations of red wine *Vitis vinifera* L. cv. Tempranillo were prepared from untreated and PL-treated grapes. Non-*Saccharomyces* yeast strains were compared with *Saccharomyces cerevisiae* (Sc) and spontaneous fermentations. The resulting wines were assessed analytically to evaluate the fermentative performance of the strains after the use of PL as a potential sanitizing technology for winemaking production to reduce the use of SO_2_.

## 2. Materials and Methods

### 2.1. Pulsed Light Treatment

**Figure 1:**
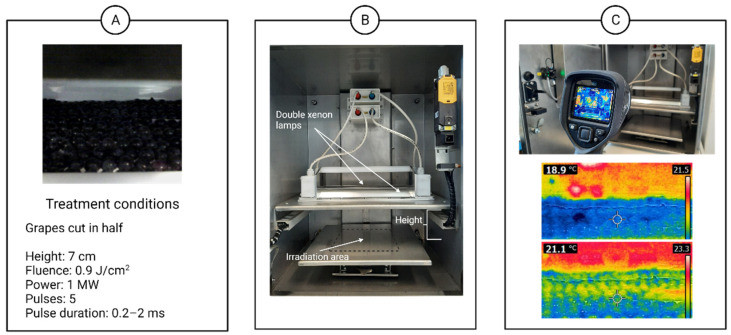
Sequence showing (**A**) interior of the PL treatment chamber; (**B**) grapes cut in half and placed under the lamps; (**C**) thermal camera used (**above**) and thermal effect (**below**).

Grapes from *Vitis vinifera* L. cv. Tempranillo from late harvest were manually destemmed, cut in half and placed on sterile plates prior to PL treatments (Figure 1A). This procedure was followed to ensure irradiation over the majority of the surface of the grapes. The grapes were handled inside a laminar flow hood wearing sterile nitrile gloves. The treatment consisted of five pulses applied with a power of 1 MW/pulse with a pulse width of 0.2 to 2 milliseconds. Therefore, the fluence achieved was 0.9 J/cm^2^. The treatment area was 25 cm long and 13 cm wide. The distance between the lamps and the sample was adjusted to 7 cm. A double xenon lamp laboratory-scale customized Claranor device was used (Figure 1B) (Claranor, Avignon, France). Treatments were performed at room temperature and a 2 °C temperature increase was observed with the use of an infrared thermal camera (Figure 1C). The temperature was measured right after the end of the treatment once the front door was opened. After treatment, the samples were manually pressed in sterile flasks and then placed in sterile 100-mL brown glass flasks for fermentation with the use of Müller valves for CO_2_ release. Untreated must was also obtained following the same aseptic procedure for comparison. All experiments were conducted in triplicate. The experiments are described as (A) control fermentations and (B) PL treatment samples.

### 2.2. Atomic Force Microscopy

The surface of treated and untreated grapes was analysed with atomic force microscopy (AFM) to determine any structural damage on the skin after the PL treatment. Topographic measurements of the skins were carried out using a Nano-Observer AFM (Concept Scientific Instruments, Les ULIS, France) operating in resonant mode. A 1-N/m rectangular silicon cantilever (model Fort, AppNano, Mountain View, CA, USA) with an 8-nm nominal tip radius was selected. Typical set point amplitudes of 4–5 volts were used during the measurements with high values of feedback and proportional and integral gains (P and I) to compensate for the high topographic variations (1–4 microns).

### 2.3. Yeast Strains and Microfermentations

The experimental design used yeast strains with different fermentative competitiveness as starters for the sequential fermentation with Sc. These strains are *Torulaspora delbrueckii* (Td) strain 1880 (CECT, Valencia, Spain), *Schizosaccharomyces pombe* (Sp) strain 938 (IFI-938, Madrid, Spain), *Metschnikowia pulcherrima* (Mp) strain MP 346 (Lallemand Bio, Madrid, Spain), *Lachancea thermotolerans* (Lt) strain L3.1 (Lallemand Bio, Madrid, Spain) and *Hanseniaspora vineae* (Hv) (Universidad de la República, Montevideo, Uruguay). Sc strain 7VA (ETSIAAB, Technical University of Madrid, Madrid) was used for pure culture fermentation and for the sequential inoculation of all previous fermentation trials. The population of the inocula used is available in Table 1. All strains were grown in YEPD media (1% yeast extract, 2% bacteriological peptone and 2% D-glucose) (Condalab, Barcelona) at a constant 25 °C for 48 h in an orbital shaker.

The fermentations were carried out in triplicate in 100-mL brown glass flasks with Müller valves for CO_2_ release. The fermentation volume was 60 mL. One millilitre of each strain was added in each flask and the time was registered as t_0_. The sequential fermentation took place at day 8 (d_8_). The fermentation kinetics were followed up by weight loss during the span of the fermentation since it relates to the amount of CO_2_ produced. The CO_2_ released was used to estimate the ethanol produced at every stage of the fermentation process (ethanol% *v/v*), and it was also useful to determine the time for the sequential fermentation to take place. The stoichiometric relation yields two molecules of ethanol and two molecules of CO_2_ from each hexose fermented. The yeasts’ populations were determined by plate counting every 48 h in YEPD agar medium (yeast extract 1%, peptone 2%, dextrose 2%, agar 1.5%) and a synthetic Lysine agar medium (Oxoid, Hampshire, UK). Total yeasts were counted on YEPD agar plates and non-*Saccharomyces* were counted on Lysine agar plates. The plates were kept at a constant 25 °C for 48 h. 

### 2.4. Oenological Parameters

The main oenological parameters (sugar concentration, concentration of titratable acids and ethanol) were measured with Fourier transform infrared spectroscopy (FTIR) analysis with a OenoFoss^TR^ apparatus (Foss Iberia, Barcelona, Spain). Samples were stirred with the use of a vortex mixer to remove any CO_2_ trapped to avoid interferences. Then, the samples were filtered with 0.45-μm cellulose methyl ester membrane filters (Phenomenex, Madrid, Spain). For the analysis, 1 mL of filtered wine was needed. The integrated software of the OenoFoss equipment provides these parameters directly. Relative accuracy is >0.95. The pH has been determined with the use of a Crison micropH 2000 pH meter. 

### 2.5. Aromatic Volatile Compounds 

Gas chromatography with a flame ionization detector (GC-FID) was used to determine volatile compounds. Samples were injected after filtration through 0.45-μm cellulose methyl ester membrane filters (Phenomenex, Madrid, Spain). An Agilent Technologies 6850 gas chromatograph (Palo Alto, CA, USA) was used. The injection temperature was 250 °C, and the detector temperature was 300 °C. A DB-624 column (60 m × 250 µm × 1.40 µm) was used with a temperature ramp of 40 °C during the first 5 min, followed by a linear increase of 10 °C per minute until 250 °C. This temperature was maintained for 5 min. The total runtime was 40 min per sample. Hydrogen was used as the carrier gas. The peak identification was possible with the retention time of the compounds using a calibration curve in accordance with the method (OIV-MA-AS315-27) [22]. The volatile compounds identified were: acetaldehyde, methanol, 1-propanol, diacetyl, ethyl acetate, 2-butanol, isobutanol, 1-butanol, acetoin, 2-methyl-1-butanol, 3-methyl-1-butanol, ethyl lactate, isobutyl acetate, 2,3-butanediol, isoamyl acetate, 2-phenylethyl acetate and 2-phenylethyl alcohol. One hundred microlitres of internal standard (4-Methyl-2-pentanol, 500 mg/L) (Fluka Chemie GmbH, Buchs, Switzerland) was added to 1-mL test samples. The limit of detection was 1 mg/L. The concentration of the volatiles is expressed as mg/L. This method is in accordance with [23].

### 2.6. Anthocyanins Quantification

The anthocyanins were identified and quantified with a series 1100 high performance liquid chromatograph (HPLC) (Hewlett-Packard, Palo Alto, CA, USA), equipped with a diode array detector (DAD). Twenty-microlitre samples of previously filtered (0.45-μm membrane) wines were injected into the HPLC apparatus. Gradients of solvents A (water/formic acid, 95:5 *v/v*) (Sigma-Aldrich GmbH, Buchs, Switzerland) and B (methanol/formic acid, 95:5 *v/v*) (Sigma-Aldrich GmbH, Buchs, Switzerland) were used in a reverse-phase Poroshell 120 C18 column (Phenomenex, Torrance, CA, USA) (50 × 4.6 mm; particle size 2.7 μm) as follows: 0–2 min, 15% B (working flow 0.8 mL/min); 2–10 min, 15–50% B linear; 10–12 min, 50% B; 12–13 min, 50–15% B linear; and 13–15 min, re-equilibration. Detection was performed by scanning in the 400–600 nm range. Quantification was performed by comparison against an external standard at 525 nm and expressed as milligram per litre of malvidin-3-O-glucoside (Extrasynthese, Genay Cedex, France) (*r^2^* = 0.9999). Anthocyanins were identified by their retention time and by comparing their UV-visible maximum absorbance. The detection limit was 0.1 mg/L. The method was adapted from [9].

### 2.7. Statistical Analysis

Statgraphics Centurion 18 software V.18.1.06 (Graphics Software Systems, Rockville, MD, USA) was used to calculate means, standard deviation and ANOVA. One-way ANOVA between groups was performed with the least significant differences (LSD). Significance was set at *p* < 0.05 for the ANOVA matrix.

## 3. Results 

**Figure 2:**
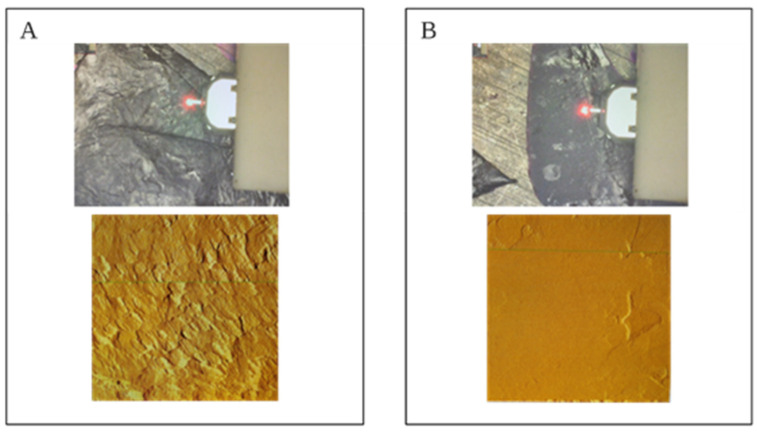
Details of the indentation done with the atomic force microscopy (AFM) on treated skin (**A**) and untreated skin (**B**) are seen in the top images, and the details on the roughness of the skin are seen on the bottom images.

In this experiment, the temperature increased 2 to 3 °C, as it was documented with a thermal camera before and after the treatment (Figure 1C). Additionally, some roughness on the skin of the berries is observed after pulses are applied (Figure 2) with the use of AFM. No severe damage was found after the treatments. 

### 3.1. Microfermentations

**Figure 3:**
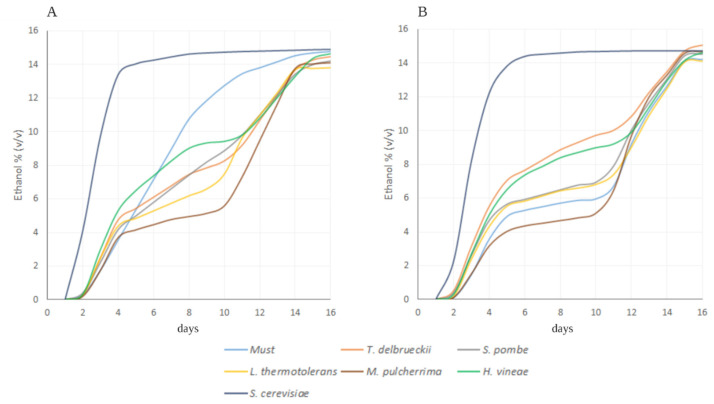
Kinetics of the alcoholic fermentation as a percentage of the estimated ethanol produced (% *v/v*) after the 2nd inoculation (sequential inoculation). (**A**) Vinifications from untreated grapes; (**B**) vinifications from PL-treated grapes.

The population of wild yeast in the pruina was reduced approximately 1.2 log from 8.8 × 10^5^ CFU/mL, found initially, to 1.1 × 10^4^ CFU/mL after the pulsed light treatment. The fermentation kinetics of musts with both treated and untreated grape are shown in Figure 3. The production of ethanol (% *v/v*) is estimated for the two weeks that the fermentations spanned as described in Section 2.3. 

The must with untreated grapes underwent a rapid uncontrolled fermentative process after a relatively slow start (Figure 3A). In the case of treated grapes, the spontaneous fermentation did not thrive until the sequential inoculation of *S. cerevisiae* strain 7VA (Figure 3B).

### 3.2. Oenological Parameters

Table 2 summarises the oenological parameters measured in all vinifications after completion. All vinifications are dry, and the concentration of sugars is below 4 g/L. The pH is significantly lower (3.5–3.6) in the fermentations with *L. thermotolerans* since the concentration of lactic acid is higher. The concentration of L-malic acid is lower, especially in the untreated vinifications, when non-*Saccharomyces* strains were inoculated.

Deviations observed in the concentration of L-lactic acid and L-malic acid are further explained in the discussion section. These deviations are more obvious in the vinifications from untreated grapes.

### 3.3. Volatile Fraction and Phenolic Profile

The volatile compounds and the phenolic profile are shown in Table 3 and Table 4, respectively. The volatile fraction was determined with the use of gas chromatography once the fermentation was completed. The results shown in Table 3 evince the influence of the different yeast strains used as starter cultures and, in the case of the PL treatment (Table 3-B), a more characteristic profile expected for each yeast species.

The TPI and the colour and the hue values observed in the vinifications after completion are shown in Table 4. In both treatments, the TPI observed was higher for the fermentations with *S. cerevisiae* with statistical significance. In addition, the hue values are above 1.5 units for all vinifications done with treated grapes.

**Figure 4:**
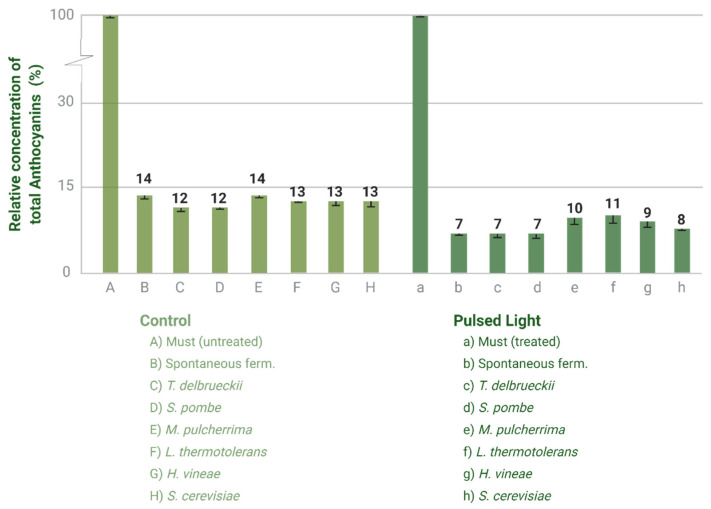
Percentage loss of anthocyanins in (**left**) wines produced with untreated grapes and (**right**) wines produced with treated grapes.

Lastly, the most remarkable difference observed in the colour profile of the wines produced is a greater decrease in anthocyanins, and therefore of colour, in the wines elaborated with treated grapes. The initial concentration of anthocyanins measured in the musts prior to the fermentation was 211.5 mg/L, and no improvement of anthocyanins extraction due to PL was detected. The anthocyanin loss during fermentation is related to the microbial counts and the fermentative process itself. The percentage loss was between 86 and 88% in the case of untreated grapes and between 89 and 93% for treated grapes (Figure 4). 

## 4. Discussion

PL is considered a nonthermal technology, as the global temperature increase at the surface of the berries after treatments is rather low. Although the temperature can increase locally up to 130 °C [24], this increase of temperature does not represent a detriment to the properties of the grape at the macro level. The roughness observed on the grape’s skin after the light pulses is perceived as scales on the surface (Figure 2A), which appear clearer than the actual skin roughness in untreated grapes (Figure 2B). The damage on the skin has been previously documented as a consequence of the disruption caused by the UV-C light component from the flashes [25]. Despite the evidence of disruption documented in previous studies, the roughness observed on the surface of the grapes may not affect the vacuoles containing anthocyanins. This does not produce an increase of anthocyanins concentration in the must, as it has been previously documented for cv. Tempranillo grapes [26].

Structural damage on the skins is then expected to increase the release of polyphenolic compounds, not only anthocyanins, during maceration in the winemaking process on a large scale. This phenomenon may be particularly important for varieties with fewer anthocyanins or wines with short maceration times.

### 4.1. Microfermentations

The reduction of counts in this experiment is smaller in comparison to reductions of about 5 to 6 log, observed with a different energy applied and different energy density [7,8]. Nonetheless, the reduction in the microbial population achieved with PL treatment affected how the selected yeast strains were implanted, and it also modulated the fermentation kinetics, as observed in Figure 3. The most noteworthy case is the spontaneous fermentation with any inoculated strain. Spontaneous fermentations are carried out in the beginning by non-*Saccharomyces* yeasts but completed by *Saccharomyces* strains whose population accounts for 10% at the 3rd day and circa 100% after the 10th day [27]. 

The phenomenon observed for the spontaneous fermentation with treated grapes was also observed in other fermentations with untreated grapes, as the initial wild population of yeasts was higher and interfered with the inoculum used. Such is the case for *S. pombe* and *T. delbrueckii*, with higher levels of ethanol produced at day 8 (Figure 3A). The case of *S. pombe* is peculiar since the consumption of sugars by this genus, both fructose and glucose, is normally slower [28]; therefore, a pure culture fermentation with this genus would take longer to be completed, whilst fermentations where this genus was inoculated together with a large amount of native yeast strains would carry on faster and in an uncontrolled manner. In general, all fermentations done with treated must seemed to have occurred at a lower rate until the sequential inoculation with *S. cerevisiae* took place. 

### 4.2. Oenological Parameters

In the same way that the kinetics of fermentation differ from untreated to treated musts during the fermentation process, the oenological parameters show differences. The fermentative strains are responsible for these differences. In this way, a lower pH was expected in the fermentations where *L. thermotolerans* was inoculated, as observed in the fermentation with treated must, and also in those fermentations where malolactic fermentation (MLF) may have taken place. MLF was prone to occurring with a higher impact in untreated musts (Table 2A), as lactic acid bacteria (LAB) have shown to be less sensitive than yeasts to the same number of PL pulses [9]. This situation can be observed in fermentations with *S. pombe* and *M. pulcherrima*, where lactic acid production, presumably produced by LAB, is enhanced in vinifications from untreated grapes. 

Regarding L-malic acid concentration, in the fermentations where *S. pombe* was used, it was expected to be consumed to almost 0 mg/L [29]. In this experiment, both fermentations had a final concentration of 0.5 g/L, but the formation of L-lactic acid suggests that the MLF took place faster and that the depletion of L-malic acid was partially carried out by bacteria and not by *S. pombe* alone. In all other fermentations where L-malic acid decreased its concentration and L-lactic acid was produced, it is deduced that LAB proliferated and thus produced L-lactic acid to different extents since, microbiology-wise, *Schizosaccharomyces* is the only genus able to reduce L-malic acid concentration efficiently [30]. 

The volatile compounds found in wines, including the volatile acids, mainly accounted for by acetic acid, are related to the grape microbiota involved in the fermentation process. Non-*Saccharomyces* yeasts usually produce volatile acidity in the range of 0.51–0.69 g/L [13]. The wines produced with untreated grapes and more uncontrolled fermentations produced higher amounts of volatile acidity in general than the treated wines (Table 2). From all the microvinifications, the untreated spontaneous one and the untreated inoculated with *S. pombe* and *M. pulcherrima* produced the highest concentrations of volatile acidity with 0.5 g/L, 0.6 g/L and 0.7 g/L, respectively. Although the species *S. pombe* can produce high values of volatile acidity at laboratory scale, usually related to its slower fermentation kinetics [31], the production of volatile acidity in these fermentations might be related to bacterial metabolism and spoilage wild yeast strains. The formation of acetic acid might be related to Indigenous Lactobacilli, the yeast genus *Hanseniaspora* and other spoilage yeasts genera, such as *Pichia*, *Candida* and *Saccharomycodes* [32]. 

Finally, although there are statistically significant differences among strains in each treatment, all fermentations were completed and produced wines with less than 4 g/L total sugars and more than 13.6% *v/v* ethanol.

### 4.3. Volatile Fraction and Anthocyanin Profile

#### 4.3.1. Aromatic Volatile’s Fraction

In a broad sense, the concentration observed for butanediol was in general higher in the fermentations carried out with PL-treated grapes, while 3-methyl-1-butanol and 2-methyl-1-butanol were generally in higher concentrations in the fermentations done with nontreated grapes. The species *Saccharomyces cerevisiae* produces high concentrations of butanediol in comparison to other non-*Saccharomyces* species [33]; thus, a better implantation in a fermentative must is expected to increase the amounts of this metabolite during fermentation. The concentration of higher alcohols has been reported to be directly proportional to the initial concentration of the inoculum [34], as was the case observed in treated grapes. Nonetheless, although the concentration of other higher alcohols is also higher with this fermentative species, in this experiment, the fermentations with nontreated grapes have given higher values in general than the PL-treated grapes. In terms of esters, the overall results do not indicate more influence towards treated and nontreated grapes except for the ethyl lactate, whose concentration tends to be higher in the nontreated grapes. In parallel, the accumulation of ethyl lactate may be closely related to the presence of lactic acid bacteria [35], which normally takes place during MLF with the conversion of L-malic acid into L-lactic acid and lactic acid producer yeasts, such as *L. thermotolerans*. Nontreated grapes are prone to having larger amounts of Indigenous strains, whilst PL-treated grapes reduce their counts and their influence in the volatile aroma profile of wines.

On the other hand, judging the microfermentations by strain, some differences may be attributed to a better implantation of yeasts after PL treatment in treated grape musts. *T. delbrueckii* has been reported to increase the concentration of aromatic compounds as fruity esters, such as isoamyl acetate, and decrease unwanted aromas, such as higher alcohols [36]. Such a decrease relates to pure culture fermentation with *Saccharomyces cerevisae*. Sequential fermentations of *T. delbrueckii* with *S. cerevisiae* yield lower values of 3-methyl-1-butanol [37].

PL-treated wines reported higher amounts of isobutyl acetate, ethyl acetate and isoamyl acetate, and lower concentrations of 2-methyl-1-butanol and 3-methyl-1-butanol. For fermentations carried out with *S. pombe*, in addition to the notorious decrease of malic acid expected, there are no particular off-flavours associated with this yeast species [28]; nonetheless, the nontreated grapes have produced wines with larger amounts of higher alcohols, especially 3-methyl-1-butanol, and considerably higher amounts of acetoin than the PL-treated grapes. Wine with PL-treated grapes had a higher concentration of ethyl lactate and ethyl acetate, which may contribute to enhancing the wine’s aroma. *L. thermotolerans* is known as a producer of variable amounts of L-lactic acid and to promote the production of 2-phenylethanol and glycerol [38]. The fermentations done with *H. vineae* are very similar in volatile compounds, except for the concentration of ethyl lactate observed in the nontreated wine. An increase of floral and fruity aromas was expected in these last wines from the production of 2-phenylethanol, as a consequence of its particular metabolism of phenylpropanoid-related compounds [39], but this was not the case. Lastly, the fermentations done with *M. pulcherrima* and *S. cerevisiae* do not have noticeable differences between treated and nontreated grapes in general, except for the higher concentration of ethyl acetate with the first species and ethyl lactate with the latter species in the treated wines. A higher concentration of esters would give wines a floral and fruity aroma and, together with other fermentative metabolites, such as higher alcohols, volatile fatty acids, carbonyl compounds and sulphur compounds, is important for the overall sensorial profile of wines [40]. 

In accordance with Lu et al. [41], spontaneous fermentations tend to increase the total amount of volatile compounds as a consequence of the proliferation of apiculate yeasts at the initial steps of the fermentation. In this last matter, *S. cerevisiae* strains used in sequential fermentations carried out with starter cultures have shown to modulate the expression of attributes observed in fermentations with non-*Saccharomyces* yeasts [42], which can be understood as a decrease of aromatic compounds, including esters, in comparison to spontaneous fermentations. This observation is valid for the spontaneous fermentations in both treatments. Finally, the use of PL has demonstrated an effect on the profile of volatile, as well as nonvolatile, compounds. Such is the case for the treatment of shiitake mushrooms to improve the synthesis of Vitamin D_2_ [43]. In this experimental set up, none of the variations observed in the volatile profile can be attributed to the use of PL with the established working conditions. Nonetheless, this effect is to be studied for larger fluences. 

#### 4.3.2. Anthocyanin’s Profile 

It is well documented that yeast strains are capable of removing anthocyanins from the fermentative media through adsorption through the cell walls [44], although the loss occurs mainly at the end of the fermentation when the cell viability is lower and the yeasts cells are permeabilised [45]. The composition of the cell walls and the polarity of the anthocyanins are two factors involved in this interaction [46]. In this regard, *Saccharomyces cerevisiae* strains had a special affinity for acyl-derivatives over nonacylated anthocyanin derivatives [44]. Differences in the amount of anthocyanins adsorbed have been recorded based on the yeast genera [46], but differences within strains from the same genus may also be expected. Other parameters involved in how anthocyanins are adsorbed into cell walls are the pH value, ethanol concentration, SO_2_ concentration and temperature [47]. In terms of the total amount of anthocyanins adsorbed by cell walls, *Saccharomyces* strains can remove between 1.6% and 5.85% from the initial pigment concentration [48]. Therefore, if the initial concentration is already low, the reduction due to adsorption would be more evident. In this experiment, the fermentations where the yeast strains had more impact on the final concentration of anthocyanins are those where the strains of *T. delbrueckii* and *S. pombe* were used. On the other hand, the fermentations carried out with strains of *M. pulcherrima* and *L. thermotolerans* retained the highest amount of anthocyanins in solution. Despite the fact that yeast strains interacted with anthocyanins during fermentation, causing colour loss by the deglycosylation of anthocyanins and pigment adsorption, other chemical interactions (oxidation, interactions with pyruvate, acetaldehyde, flavanols, condensed tannins, etc.) may have caused an even larger reduction of anthocyanins and, as a consequence, of total phenolic compounds [45]. Due to the small fermentation volume used, the skins were removed after the grapes were crushed, and the pigment’s extraction was limited. The reduction of anthocyanins is more evident in small fermentation volumes without skin maceration. 

Another consequence of the higher interaction of the strains in the treated must is the higher values obtained for the hue (Table 4). This can be explained by a higher loss of acyl-derivatives, promoting the reduction of blue colour and increasing the yellow fraction in wines [44]. Acetylated anthocyanins in particular are very important for predicting the colour of wines noticeable by the naked eye [49]. The values for CI correspond to the concentration of anthocyanins measured and are therefore higher for the wines produced from the untreated must, as are the total polyphenolic index (TPI) values. Occasionally, *Saccharomyces cerevisiae* has less β-glucosidase strain-related activity than non-*Saccharomyces* yeasts in spontaneous and inoculated fermentations [50]. Moreover, this strain has also rapider fermentation kinetics. These two features may have resulted in higher TPI and CI values.

## 5. Conclusions

The use of PL to reduce the Indigenous yeast strains in late harvest grapes has demonstrated its suitability for the implantation of selected yeast strains, in particular for the use of *Lachancea thermotolerans*, which is interesting for the increase of L-lactic acid during winemaking in warm areas. Starter cultures have better options to thrive once the native strains reduce their counts without the use of SO_2_. Nonetheless, the energy density used for the pretreatment should be higher, or the number of pulses larger, to avoid the opportunity for bacteria to thrive and coferment with the starter culture. Low fluence treatments, below 1 J/cm^2^, may be prone to poorly reducing microbial counts. The drawback observed at laboratory scale regarding colour loss should not be a problem on a large scale where it is possible to have musts with a proper maceration time; however, even so, the higher loss observed in wines produced with treated must calls for a larger number of counts and a better implantation. The efficiency of this nonthermal technology is yet to be tested on a large scale in liquids with the opacity, particle distribution, and density of wine must as a pretreatment after crushing to reduce the use of SO_2_ as much as possible. Finally, other effects of the use of PL to be considered include the stability of colour and the organoleptic properties of wine over time. 

## Figures and Tables

**Table 1:** Population determined with plate counting for each inoculum. The two first columns indicate the inocula used as fermentation starter (d_0_), and the third column refers to the inoculum used for the sequential fermentation with Sc (d_8_).

Starter	Inocula d_0_ CFU/mL	Sequential Inocula d_8_ CFU/mL
*T. delbrueckii*	1.1 × 10^8^	Sc 6.0 × 10^7^
*S. pombe*	3.2 × 10^7^	Sc 6.0 × 10^7^
*M. pulcherrima*	6.9 × 10^7^	Sc 6.0 × 10^7^
*L. thermotolerans*	6.0 × 10^7^	Sc 6.0 × 10^7^
*H. vineae*	1.3 × 10^8^	Sc 6.0 × 10^7^
*S. cerevisiae*	1.0 × 10^8^	Sc 6.0 × 10^7^

**Table 2:** Oenological parameters determined with Fourier transform infrared spectroscopy (FTIR) for (A) control fermentations with untreated must and (B) Pulsed light irradiation (PL) treatment. Average and standard deviation (STD) for *n* = 3. Different letters indicate statistical difference within each treatment *p* > 0.05.

	Ethanol	pH	Total Acidity ^1^	Volatile Acidity ^2^	Malic Acid	Lactic Acid	Fructose	Glucose
	% *v/v*		g/L	g/L	g/L	g/L	g/L	g/L
A								
Spontaneous	14.1 ± 0.1 a	4.0 ± 0.0 a	4.4 ± 0.1 b	0.5 ± 0.0 bc	0.7 ± 0.1 c	1.3 ± 0.1 bc	2.7 ± 0.1 ab	0.0 ± 0.0 b
*T. delbrueckii*	14.1 ± 0.1 a	3.9 ± 0.0 a	4.3 ± 0.2 b	0.1 ± 0.0 d	1.3 ± 0.0 b	0.1 ± 0.0 c	2.6 ± 1.0 ab	0.4 ± 0.2 b
*S. pombe*	13.7 ± 0.3 b	4.0 ± 0.1 a	5.5 ± 1.6 ab	0.6 ± 0.1 b	0.5 ± 0.2 c	1.9 ± 0.9 bc	1.3 ± 1.5 bc	1.0 ± 0.8 b
*M. pulcherrima*	13.7 ± 0.3 b	3.9 ± 0.0 a	6.8 ± 1.3 a	0.9 ± 0.3 a	0.1 ± 0.1 d	3.1 ± 0.8 ab	0.3 ± 0.3 c	2.2 ± 1.3 a
*L. thermotolerans*	13.8 ± 0.2 ab	3.6 ± 0.2 b	6.9 ± 1.1 a	0.4 ± 0.0 bc	0.2 ± 0.3 d	4.6 ± 1.7 a	0.0 ± 0.1 c	0.1 ± 0.2 b
*H. vineae*	14.2 ± 0.1 a	3.9 ± 0.0 a	4.1 ± 0.1 b	0.3 ± 0.0 cd	1.4 ± 0.1 b	0.0 ± 0.0 c	3.0 ± 0.6 a	0.9 ± 0.3 b
*S. cerevisiae*	13.8 ± 0.2 ab	3.9 ± 0.0 a	5.6 ± 0.1 ab	0.2 ± 0.0 cd	2.2 ± 0.1 a	0.7 ± 0.2 c	0.5 ± 0.1 c	0.0 ± 0.0 b
B								
Spontaneous	14.1 ± 0.1 a	3.9 ± 0.0 c	4.8 ± 0.0 c	0.4 ± 0.1 ab	1.6 ± 0.0 b	0.0 ± 0.0 b	0.9 ± 0.1 b	0.1 ± 0.1 b
*T. delbrueckii*	13.6 ± 0.2 cd	3.9 ± 0.0 bc	4.6 ± 0.1 c	0.2 ± 0.1 d	1.3 ± 0.1 bc	0.2 ± 0.1 b	1.7 ± 0.6 a	0.3 ± 0.3 a
*S. pombe*	13.8 ± 0.2 bc	4.0 ± 0.0 a	3.6 ± 0.2 d	0.3 ± 0.0 c	0.5 ± 0.1 d	0.5 ± 0.1 b	0.9 ± 0.2 b	0.2 ± 0.1 ab
*M. pulcherrima*	13.4 ± 0.2 d	3.9 ± 0.0 b	4.7 ± 0.3 c	0.4 ± 0.0 a	0.0 ± 0.1 e	0.8 ± 0.1 b	0.0 ± 0.0 d	0.3 ± 0.1 a
*L. thermotolerans*	13.7 ± 0.1 bc	3.5 ± 0.0 d	7.2 ± 0.5 a	0.3 ± 0.0 bc	1.1 ± 0.6 c	4.9 ± 0.7 a	0.7 ± 0.2 bc	0.0 ± 0.0 b
*H. vineae*	13.8 ± 0.1 ab	3.9 ± 0.0 c	4.4 ± 0.1 c	0.3 ± 0.0 bc	1.3 ± 0.1 bc	0.2 ± 0.1 b	1.0 ± 0.1 b	0.1 ± 0.1 b
*S. cerevisiae*	13.6 ± 0.1 cd	3.9 ± 0.0 c	5.8 ± 0.1 b	0.2 ± 0.0 d	2.1 ± 0.1 a	0.7 ± 0.0 b	0.3 ± 0.1 cd	0.0 ± 0.0 b

^1^ Expressed as tartaric acid; ^2^ Expressed as acetic acid.

**Table 3:** Volatile fraction, in mg/L, determined with flame ionization detector (GC-FID). The results correspond to (A) control fermentations and (B) PL treatment. Average and STD for *n* = 3. Different letters indicate a significant difference within the same treatment for each compound.

A	Spontaneous	*T. delbrueckii*	*S. pombe*	*L. thermotolerans*	*H. vineae*	*M. pulcherrima*	*S. cerevisiae*
Acetaldehyde	7.8 ± 0.9 a	12.6 ± 8.4 a	13.1 ± 3.3 a	9.4 ± 1.0 a	8.4 ± 0.5 a	7.9 ± 0.3 a	7.1 ± 1.2 a
Methanol	26.9 ± 1.0 ab	26.6 ± 4.2 b	23.0 ± 0.3 ab	23.1 ± 1.2 b	24.9 ± 3.7 b	23.7 ± 1.7 b	29.7 ± 2.0 a
1-propanol	43.2 ± 4.0 b	52.2 ± 1.3 a	42.6 ± 1.2 b	44.6 ± 1.6 b	40.3 ± 4.6 b	41.2 ± 1.6 b	20.1 ± 1.0 c
Diacetyl	1.5 ± 0.1 ab	1.5 ± 0.1 b	1.7 ± 0.1 ab	1.6 ± 0.1 ab	1.9 ± 0.2 a	1.6 ± 0.1 b	1.7 ± 0.4 ab
Ethyl acetate	159.8 ± 17.9 a	82.2 ± 16.2 c	90.9 ± 10.3 c	62.9 ± 7.6 d	80.8 ± 1.4 c	113.1 ± 7.4 b	19.8 ± 1.5 e
2-butanol	n.d.	n.d.	n.d.	n.d.	n.d.	n.d.	n.d.
Isobutanol	45.0 ± 7.6 ab	56.9 ± 12.9 a	39.8 ± 3.2 b	38.9 ± 5.1 b	23.4 ± 1.7 c	42.6 ± 5.7 b	27.0 ± 0.5 c
1-butanol	4.3 ± 0.2 a	4.8 ± 0.5 a	4.4 ± 0.3 a	4.6 ± 0.3 a	4.9 ± 5.1 a	4.8 ± 0.1 a	4.8 ± 0.1 a
Acetoin	6.0 ± 0.5 b	5.4 ± 0.4 b	19.2 ± 10.1 a	6.2 ± 0.7 b	7.3 ± 1.4 b	7.1 ± 1.0 b	5.8 ± 0.3 b
3-methyl-1-butanol	102.5 ± 4.7 c	139.4 ± 18.1 b	95.2 ± 6.3 c	113.0 ± 7.0 bc	106.4 ± 13.1 c	93.6 ± 5.0 c	192.4 ± 37.5 a
2-methyl-1-butanol	47.4 ± 2.6 a	45.2 ± 2.7 a	45.2 ± 1.2 a	38.7 ± 3.2 a	35.2 ± 1.9 a	47.0 ± 2.8 a	64.8 ± 56.1 a
Isobutyl acetate	1.1 ± 1.5 a	1.1 ± 1.9 a	1.0 ± 1.7 a	2.3 ± 0.9 a	1.6 ± 2.8 a	3.3 ± 0.8 a	3.0 ± 0.6 a
Ethyl butyrate	0.8 ± 1.1 ab	3.0 ± 2.4 a	0.9 ± 0.7 b	1.8 ± 0.9 ab	0.9 ± 0.8 ab	0.9 ± 0.8 ab	1.4 ± 0.4 ab
Ethyl lactate	46.5 ± 8.8 ab	31.9 ± 17.0 bc	13.7 ± 4.5 d	21.8 ± 8.3 cd	51.2 ± 6.6 a	15.6 ± 1.5 d	11.6 ± 1.2 d
2,3-butanediol	529.2 ± 17.4 bcd	547.4 ± 67.8 bc	672.6 ± 35.7 a	586.7 ± 63.5 b	470.2 ± 53.7 cd	614.5 ± 33.4 ab	445.9 ± 24.4 d
Isoamyl acetate	1.9 ± 0.1 abc	2.7 ± 0.3 a	1.8 ± 0.1 bc	2.5 ± 0.1 ab	1.9 ± 0.1 ab	1.9 ± 0.0 b	1.2 ± 1.0 c
Hexanol	4.1 ± 0.8 b	4.4 ± 0.1 b	4.6 ± 0.1 ab	4.4 ± 0.1 b	4.3 ± 0.1 b	4.6 ± 0.1 ab	5.0 ± 0.6 a
2-phenyl ethanol	29.2 ± 0.8 cd	35.7 ± 2.8 bc	29.8 ± 1.2 cd	41.5 ± 7.3 b	25.6 ± 1.5 d	34.5 ± 2.3 bc	76.6 ± 6.5 a
2-phenyl ethyl acetate	6.5 ± 1.5 a	5.3 ± 0.2 a	5.1 ± 0.1 a	5.0 ± 0.3 a	6.2 ± 1.9 a	5.2 ± 0.3 a	5.2 ± 0.4 a
B	Spontaneous	*T. delbrueckii*	*S. pombe*	*L. thermotolerans*	*H. vineae*	*M. pulcherrima*	*S. cerevisiae*
Acetaldehyde	14.1 ± 0.9 a	10.0 ± 1.2 b	10.0 ± 3.0 b	8.2 ± 1.8 b	13.5 ± 0.7 a	7.8 ± 1.2 b	8.4 ± 0.1 b
Methanol	21.7 ± 1.2 ab	24.0 ± 2.0 a	24.0 ± 3.5 a	22.4 ± 3.3 ab	17.9 ± 1.3 b	26.0 ± 0.2 a	22.5 ± 3.6 a
1-propanol	54.0 ± 1.2 bc	71.5 ± 5.0 a	40.6 ± 15.1 c	52.8 ± 1.3 b	48.7 ± 2.2 bc	40.5 ± 7.1 c	26.6 ± 0.6 d
Diacetyl	1.5 ± 0.1 b	1.7 ± 0.3 ab	1.6 ± 0.1 ab	1.5 ± 0.1 b	1.8 ± 0.1 a	1.5 ± 0.0 b	1.5 ± 0.1 b
Ethyl acetate	147.3 ± 3.2 a	118.5 ± 17.8 b	120.3 ± 3.2 b	96.5 ± 4.6 c	81.2 ± 3.2 c	134.1 ± 16.3 ab	24.6 ± 2.8 d
2-butanol	n.d.	n.d.	n.d.	n.d.	n.d.	n.d.	n.d.
Isobutanol	64.4 ± 2.7 a	53.5 ± 6.1 ab	39.2 ± 6.5 c	42.2 ± 11.0 c	19.5 ± 0.4 d	45.3 ± 2.6 bc	25.5 ± 0.8 d
1-butanol	4.4 ± 0.1 bc	4.5 ± 0.2 abc	4.4 ± 0.5 bc	4.9 ± 0.4 a	4.5 ± 0.1 abc	4.1 ± 0.1 c	4.8 ± 0.1 ab
Acetoin	4.8 ± 0.2 a	7.2 ± 1.4 a	8.0 ± 3.9 a	7.1 ± 1.5 a	8.4 ± 2.5 a	6.3 ± 0.7 a	5.8 ± 1.0 a
3-methyl-1-butanol	89.5 ± 3.2 cd	132.9 ± 16.4 b	64.2 ± 14.8 e	113.0 ± 16.5 bc	100.0 ± 4.6 cd	80.6 ± 14.2 de	167.3 ± 3.0 a
2-methyl-1-butanol	51.2 ± 0.3 b	44.1 ± 4.5 bc	33.3 ± 7.9 de	36.4 ± 8.7 cd	25.4 ± 1.0 e	31.4 ± 2.7 de	81.5 ± 1.9 a
Isobutyl acetate	2.6 ± 0.1 ab	3.1 ± 0.9 a	2.3 ± 1.0 ab	1.0 ± 0.9 b	1.8 ± 1.8 ab	3.2 ± 0.9 a	0.8 ± 1.5 b
Ethyl butyrate	2.7 ± 0.1 a	1.3 ± 0.0 ab	1.1 ± 1.8 ab	1.4 ± 0.2 ab	0.5 ± 0.8 b	1.1 ± 1.1 ab	1.6 ± 0.5 ab
Ethyl lactate	36.1 ± 2.2 a	25.6 ± 16.2 a	31.2 ± 3.2 a	42.2 ± 3.9 a	22.5 ± 8.1 a	12.2 ± 0.7 a	28.7 ± 13.3 a
2,3-butanediol	766.5 ± 8.5 a	588.3 ± 22.0 bc	775.9 ± 182.3 a	687.5 ± 73.7 ab	459.7 ± 38.1 c	638.7 ± 54.0 ab	596.7 ± 41.4 bc
Isoamyl acetate	1.7 ± 0.0 cd	8.2 ± 0.4 a	1.7 ± 0.0 d	2.3 ± 0.6 bc	2.3 ± 0.3 b	1.8 ± 0.1 bcd	2.0 ± 0.1 bcd
Hexanol	4.2 ± 0.0 a	3.8 ± 0.0 b	4.1 ± 0.2 ab	3.9 ± 0.2 ab	4.1 ± 0.2 ab	4.1 ± 0.1 a	4.1 ± 0.2 ab
2-phenyl ethanol	24.6 ± 1.6 cd	35.2 ± 8.1 b	20.5 ± 4.1 d	29.6 ± 0.9 bc	25.5 ± 2.0 cd	24.1 ± 2.6 cd	62.9 ± 2.1 a
2-phenyl ethyl acetate	5.5 ± 0.1 a	5.5 ± 0.4 a	6.3 ± 2.1 a	4.2 ± 3.8 a	6.6 ± 0.8 a	6.0 ± 1.5 a	5.1 ± 0.1 a

n.d.—no-data below detection limit.

**Table 4:** Total polyphenolic index (TPI), colour intensity (CI) and hue for wines produced from (A) control fermentations and (B) PL treatment. Average and STD for *n* = 3. Different letters indicate significant differences within the same treatment.

	TPI	CI	Hue
A			
Spontaneous	11.1 ± 0.1 bc	0.8 ± 0.0 b	1.2 ± 0.0 bc
*T. delbrueckii*	10.4 ± 0.6 c	0.6 ± 0.1 c	1.3 ± 0.1 b
*S. pombe*	11.2 ± 0.2 bc	0.7 ± 0.0 bc	1.4 ± 0.0 a
*M. pulcherrima*	10.7 ± 0.1 bc	0.8 ± 0.0 b	0.9 ± 0.0 d
*L. thermotolerans*	11.3 ± 0.3 b	0.8 ± 0.0 b	1.3 ± 0.0 b
*H. vineae*	10.9 ± 0.1 bc	0.6 ± 0.0 c	1.3 ± 0.0 ab
*S. cerevisiae*	13.0 ± 0.1 a	1.0 ± 0.0 a	1.1 ± 0.0 cd
B			
Spontaneous	8.7 ± 0.0 b	0.6 ± 0.0 a	2.0 ± 0.0 ab
*T. delbrueckii*	8.2 ± 0.1 b	0.3 ± 0.0 c	2.6 ± 0.1 a
*S. pombe*	8.9 ± 0.1 b	0.5 ± 0.1 ab	2.0 ± 0.3 ab
*M. pulcherrima*	8.4 ± 0.3 b	0.4 ± 0.1 abc	2.1 ± 0.1 ab
*L. thermotolerans*	8.5 ± 0.4 b	0.3 ± 0.1 bc	1.5 ± 0.1 b
*H. vineae*	8.7 ± 0.2 b	0.3 ± 0.1 c	2.4 ± 0.5 a
*S. cerevisiae*	10.7 ± 0.9 a	0.4 ± 0.0 abc	1.5 ± 0.1 b

## Data Availability

No new data were created or analysed in this study. Data sharing is not applicable to this article.
